# Comb Irradiation Has Limited, Interactive Effects on Colony Performance or Pathogens in Bees, *Varroa destructor* and Wax Based on Two Honey Bee Stocks

**DOI:** 10.3390/insects10010015

**Published:** 2019-01-08

**Authors:** Lilia I. de Guzman, Michael Simone-Finstrom, Amanda M. Frake, Philip Tokarz

**Affiliations:** Honey Bee Breeding, Genetics and Physiology Laboratory, USDA-ARS, Baton Rouge, LA 70820, USA; mandy.frake@ars.usda.gov (A.M.F.); philip.tokarz@ars.usda.gov (P.T.)

**Keywords:** gamma irradiation, mite-resistant honey bees, wax-borne pathogens, integrated pest management

## Abstract

Parasitic mites and pathogens compromise honey bee health. Development of sustainable and integrative methods of managing these problems will minimize their detrimental impact on honey bees. Here, we aimed to determine if the combination of using mite-resistant stocks along with gamma-irradiated combs influences colony health and productivity. The major finding concerned honey bee genotype confirming that Russian honey bees are more resistant to *Varroa destructor* than Italian honey bees. The effect of comb irradiation was inconsistent showing a significant increase in adult bee population and amount of stored pollen in 2015, but not in 2016. The increased amount of stored pollen was probably associated with larger adult population in colonies with irradiated combs in September 2015 regardless of honey bee stock. Nevertheless, the ability of bees to collect and store more pollen in the irradiated group does not appear to compensate the negative impacts of mite parasitism on honey bees especially in the Italian bees, which consistently suffered significant colony losses during both years. Results of viral analyses of wax, newly emerged bees, and *Varroa* and their pupal hosts showed common detections of Deformed wing virus (DWV), Varroa destructor virus (VDV-1), Chronic bee paralysis virus (CBPV), and Black queen cell virus (BQCV). Wax samples had on average ~4 viruses or pathogens detected in both irradiated and non-irradiated combs. Although pathogen levels varied by month, some interesting effects of honey bee stock and irradiation treatment were notable, indicating how traits of mite resistance and alternative treatments may have additive effects. Further, this study indicates that wax may be a transmission route of viral infection. In addition, pupae and their infesting mites from Italian colonies exhibited higher levels of DWV than those from Russian colonies suggesting potential DWV resistance by Russian honey bees. CBPV levels were also reduced in mites from Russian colonies in general and in mites, mite-infested pupae, and newly emerged bees that were collected from irradiated combs. However, BQCV levels were not reduced by comb irradiation. Overall, the contribution of irradiating comb in improving honey bee health and colony survival appears to be subtle, but may be useful as part of an integrated pest management strategy with the addition of using mite-resistant stocks.

## 1. Introduction

Loss of honey bee colonies and reduction of colony productivity are the major concerns for beekeepers around the world. Parasitic mites and pathogens are devastating to honey bee health [1], and controlling them is a recurring issue of great significance to the bee industry. While acaricides, fungicides and antibiotics are readily available and oftentimes used to mitigate these problems, they pose risks to honey bees and can contaminate hive products [2]. Improved management practices can reduce or eliminate complete reliance on synthetic pesticides by beekeepers, particularly through the use of integrated pest management (IPM). Here, we are specifically addressing mite-resistant stocks and the potential of combining resistance traits of the bees with management strategies using gamma-irradiated combs, a treatment used to reduce the spread of pathogens when reusing combs and swapping among hives. 

Breeding-based strategies for managing *Varroa destructor* have been demonstrated through various studies (e.g., [3,4,5,6]). Mite population growth is slow in resistant stocks [3,7], delaying the onset of treatment threshold [8] or the frequency of treatment applications. Nonetheless, the benefits of using mite-resistant stocks can be maximized when used with other approaches. While drone brood removal [9] and use of screen bottom boards [8] can be important components of an IPM program for regulating *Varroa* populations, some attributes of combs are also reported to be helpful. Small cell size contributes to the resistance to *Varroa* displayed by Africanized bees (AHBs) in South America [10,11]. However, this resistance was not observed in the South African honey bees (*Apis mellifera scutellata*), which also build combs with similar cell size [12]. Accordingly, the slow *Varroa* population growth in *A m. scutellata* was largely due to suppression of mite reproduction [13], an observation made by Harbo and Harris [14] in European honey bees (EHB), which build larger cells than Africanized or African bees. When compared to AHBs in South America, EHBs supported higher *Varroa* prevalence [11]. Nevertheless, different stocks of EHBs vary in their response to *Varroa* infestations [5,6]. Lower number of viable female daughters was recorded in combs built by Russian honey bees than in combs built by Italian honey bees [15]. Prevalence of *Varroa* has also been found to be generally higher in old combs, with the exception that in Russian honey bee colonies the number of viable female offspring produced per reproductive cycle is low, regardless of comb age [15].

Combs are repeatedly used by honey bees either for food storage or brood rearing. Due to the accumulation of cocoons through years of brood rearing [16], the size of cells is greatly reduced [17]. Simultaneously, traces of propolis, pollen, and fecal matters are entrapped [18]. Due to the lipophilic nature of wax, contaminants that are detrimental to honey bees’ health including pesticides [19] and various pathogens such as *Paenibacillus larvae* (American foulbrood, AFB) [20] are also absorbed. Unfortunately, these pathogens in combs linger as dormant spores and thrive when the optimum conditions are reached [21]. A preliminary study showed that viruses can also be wax-borne [22], though a systematic analysis of viral loads in wax has not been done prior to this study.

Replacement of combs is considered a sanitary measure in Europe [23] and Asia (per. obs.). Although new combs can optimize overall health and performance of honey bee colonies [24], beekeepers in the United States reuse old combs because of its cost-effectiveness. In addition, secretion of wax to draw foundations by bees requires a large amount of energy [25], which reduces honey production [26], and slows down brood production. The spread of pathogens and parasites is largely facilitated by this common practice, which can be prevented by gamma irradiation [27,28,29] in some cases. In a recent survey, beekeepers who reused old combs lost on average 4.7 more colonies per 100 managed colonies than those who did not [30]. However, the survey failed to identify comb features that may have influenced short survival. The arrival of *V. destructor*, *Acarapis woodi*, *Aethina tumida* and *Nosema ceranae*, the increased presence of mite-borne viruses, and the widespread use of pesticides exacerbate the potential challenges not only for worker bees, but also for queens and drones [31,32]. 

Gamma irradiation is being used to inactivate pathogens infecting various commodities and equipment including beekeeping-related materials to control AFB [28,29,33]. Recently, it has been demonstrated that gamma irradiation can effectively inactivate *Ascosphaera apis*, *Nosema* and Deformed wing virus (DWV), and only partially inactivate Black queen cell virus (BQCV) and Chronic bee paralysis virus (CBPV) [34]. Most recent studies on gamma irradiation have focused on its effect on the growth and infectivity of these pathogens in the laboratory or on reducing pathogens in pollen [35,36]. Recently, it has been shown that worker bees reared in irradiated combs had lower levels of DWV, but its effect diminished through the summer season [37]. Nonetheless, the same study showed that honey bee stock, but not comb-irradiation influences foraging activities of workers suggesting that any effects of irradiation on viral levels may not translate to colony level effects on productivity. In a preliminary study that evaluated re-used combs from colonies that suffered from Colony Collapse Disorder (CCD), bee packages installed in colonies with irradiated combs were stronger than those that received non-irradiated combs [27]. However, wax-borne pathogens, particularly viruses, were not analyzed. Given these purported benefits of gamma irradiation, we explored its use particularly as part of an IPM program. The interactive effects of mite-resistant stocks and the comb treatment, both of which are known to impact viral levels, was also investigated. In addition, several novel secondary questions were evaluated including how stock and comb treatment influenced pathogen levels in wax, bees and mites. 

## 2. Materials and Methods 

### 2.1. Establishing Test Colonies 

During each spring of 2015 and 2016, 40 packages (1.4 kg) were established near Baton Rouge, Louisiana using the large package technique [37] obtained by shaking bees from untreated colonies of pure Russian and several hybrids of Italian and Russian colonies from an earlier experiment. This mixture of bees allowed the establishment of colonies with uniform bees and mites at the beginning of each experiment. In both years, each colony was randomly assigned to one of the four treatments: (1) Italian honey bee queen with irradiated combs (10 colonies); (2) Italian honey bee queen with non-irradiated combs (Control, 10 colonies); (3) Russian honey bee queen with irradiated combs (10 colonies); and (4) Russian honey bee queen with non-irradiated combs (Control, 10 colonies). Each colony was housed in 10-frame Langstroth medium boxes. Test combs were a mixture of old (had been used in brood rearing) and new (never been used in brood rearing) combs. Irradiated combs and beekeeping equipment were exposed to 25 Kgy using a cobalt-60 gamma irradiator (2015 at Food Technology Service, Inc., Mulberry, FL, USA; 2016 at Gateway America, Gulfport, MS, USA). For both years of experiment, the naturally-mated Italian queens used were purchased from the same commercial queen breeder in California, while the Russian queens were naturally mated locally [37]. Colonies that had a queen change during the course of the experiment were excluded from analyses. 

### 2.2. Measuring Colony Performance and Mite Infestation 

For both years of observations, colony evaluations commenced when test bees populated the hives ~6 weeks after queen introduction. Colony evaluations were conducted twice in 2015 (June and September) and 3 times in 2016 (June, August and October). Overwintering survival during the following year was also recorded. For each colony, the numbers of sealed or unsealed worker brood cells were determined by visual estimation of comb area covered by capped or uncapped worker brood [38]. The same technique was also used for estimating the number of cells containing pollen. Adult bee population was estimated by visual estimation of the percentage of a comb occupied by adult bees [39]. *Varroa* infestation parameters were assessed using two frames of brood containing purple-eyed or older pupae. Brood cells were examined until either 30 infested cells were obtained or 200 brood cells were examined per colony per sampling time. At high levels of infestation, pulling more brood beyond 30 infested cells does not contribute significantly in the evaluation of infestation parameters. The percentage of *Varroa* infestation on adult bees was also estimated by collecting ~300–400 bees from at least two brood frames, washing bees using detergent and counting the mites and bees [40,41]. The number of mites in each colony was derived from the numbers of adult female mites (foundress and daughters) in infested cells and mites on adult bees, numbers of capped brood and adult bees following established protocols [42]. In brief, mites in the brood were estimated by dividing the total number of adult females (foundress + daughters) found in infested cells by the number of cells examined, and then multiplying it by the total number of capped brood in the colony. Similarly, mites on adult bees were estimated by dividing the number of mites by the number of bees sampled, and multiplying it by the total number of adult bees in the colony. Numbers of mites in the brood and on adult bees were then added to estimate the total number of mites in a colony. 

### 2.3. Pathogen Load Analyses 

Marked irradiated or non-irradiated combs were introduced to their respective treatment groups (irradiated and control). Test combs were moved towards the middle or next to brood frames. Wax sampling was conducted in June, August and October 2016 by taking three cells from different areas of each test comb. If these combs had emerging brood at the time of wax sampling, 12 newly emerged bees were also collected per comb. In addition, purple-eyed pupae and their infesting mite were collected in October 2016.

Samples were analyzed via qRT-PCR for bacterial (*Melissococcus plutonius*, the causative agent of European foulbrood [EFB]; *Paenibacillus larvae*, the causative agent of American foulbrood [AFB]), fungal (*Ascosphaera apis*, the causative agent of chalkbrood [CB]), microsporidian (*Nosema* spp.)), and viral pathogens (the Acute bee paralysis-Kashmir bee-Israeli acute paralysis virus complex [AKI], Black queen cell virus [BQCV], Chronic bee paralysis virus [CBPV], Deformed wing virus [DWV], Lake Sinai virus [LSV-U], and Varroa destructor virus-1 [VDV-1]). VDV-1 is a DWV-like virus that has been found to have recently spread across the US [43]. While it is referred to as DWV-B in some cases, here we refer to it as VDV-1 to be consistent with the International Committee for the Taxonomy of viruses [44,45]. Mites and pupae were only analyzed for viral loads, while wax and newly emerged bees were tested using the full pathogen panel. In addition, *Pros54* (reference gene) and vitellogenin (*Vg*) analyses were performed for the newly emerged bees as a general measure of bee health. 

Total RNA from wax, mites and bees was extracted and analyzed following previously established and adaptations of standard protocols [34,37] (see Table S1 for specific primers used). In brief, RNA extractions were completed using the Maxwell^®^ 16 LEV simplyRNA system (Promega, Madison, WI, USA). Wax samples were prepared by pooling equal amounts of the three cells in 200 µL EtOH at 100% and 300 µL Homogenation Solution (Promega). Single mite extractions were accomplished in 50 µL sterile 1X PBS with 1% Lysis Buffer (Promega), then brought to 300 µL using Homogenation Solution (Promega). Pupae and newly emerged bees were extracted individually using 200 µL each of Homogenation Solution and Lysis Buffer (Promega). Pooled bee samples were first processed frozen using Omni BeadRuptor-24, then adding 2.5 mL sterile 1X PBS. A volume of 400 µL was then mixed with equal volume of Homogenation Solution (Promega). Up to 400 µL of each extract was placed in LEV cartridges and RNA extraction was completed using standard simplyRNA protocol. Samples were quantified using NanoDrop One (Thermo Fisher Scientific, Waltham, MA, USA) and standardized at 250 ng/µL in sterile nuclease-free H_2_O (Promega) prior to cDNA synthesis.s

cDNA template was generated from 250 ng of total RNA using the QuantiTect Reverse Transcription Kit (Qiagen, Venlo, Netherlands), following the manufacturer’s instructions. qPCR for BQCV, CBPV and DWV was performed in triplicate with a QuantStudio™ 6 Flex System (Applied Biosystems, Foster City, CA, USA) using 2 µL of cDNA template per 20 µL reaction containing 1X PowerUP™ SYBR^®^ Green (Thermo Fisher Scientific). The thermal program for the reactions was a hold period consisting of 2 min at 50 °C then 2 min at 95 °C, followed by 40 cycles of 95 °C for 15 s, 59 °C for 20 s, and 72 °C for 30 s. DWV analysis utilized a 53.5 °C annealing temperature. At the end of the PCR reaction, a melt-curve dissociation analysis was performed to confirm product size for DWV, BQCV and CBPV. Virus titers were determined using standard curves of plasmid standards (generated by GeneArt, Invitrogen, Carlsbad, CA, USA). Linearized plasmid standards, containing from 10^12^ to 10^5^ copies per reaction, were used as templates to assess primer efficiency and quantify the relative amount of virus following standard practices [34]. Linear standard equations were generated using the log_10_ of the initial plasmid copy number, and this was used to determine the virus copy number. All other target analysis (Pros54, *Vg*, AKI, LSV-U, VDV-1, *Nosema* spp., *A. apis*, EFB, and *P. larvae*) was performed in triplicate using CFX Connect (Bio-Rad, Hercules, CA, USA) using 1 µL cDNA template per 10 µL reaction containing 1X Sso Universal SYBR^®^ Green (Bio-Rad). Thermal protocol consists of a hold period consisting of 2 min at 95 °C, followed by 40 cycles of 95 °C for 5 s and 59 °C for 5 s. A melt-curve dissociation analysis performed to confirm product size. Following previous studies [46,47], due to different materials with unreliable reference genes, transcripts for AKI, *P. larvae*, EFB, *A. apis*, LSV-U, *Nosema* and VDV-1 were evaluated by C_t_ values, based on the assumption that the amount of template after quantification and appropriate dilution did not differ systematically among treatment groups. Tests of technical error indicated little variation in C_t_ values within a sample. Relative *Vg* was calculated using the ΔΔC_t_ method following standard protocols and log-transformed for analysis to conform to normality [48]. Viral copy numbers, when determined, were also log-transformed. 

For *Nosema* spore count, 25 bees were pooled and macerated using standard protocol [49].

### 2.4. Data Analyses 

To better approximate normality, data on bee population were square-root transformed and data on percentage mite infestation were arc-sine square-root transformed prior to analyses. First, a three-factor repeated measures analysis was performed to determine the effects of honey bee stock, comb treatment, and date of observation on bee population and mite infestation parameters with colony as the repeated subject to control for colony variation. There were no three-way interactions among date of observation, honey bee type and comb treatment detected (see Table S2). Hence, a two-factor fixed effect model was then used to determine the effects of comb treatment and honey bee stock on mite population in the colonies. Pairwise post-hoc *t*-tests were conducted to determine specific significant differences when appropriate. For each year of observation, colony survival was analyzed using PROC LIFETEST (SAS v9.4). 

For the pathogen analyses, a three-factor repeated measures analysis was performed to determine the effects of stock, comb treatment, and sampling date on pathogen levels in wax. A three-factor analysis of variance (ANOVA) was used to analyze the effects of stock, comb treatment and sampling date on the pathogen loads in newly emerged bees. A two-way ANOVA was used to determine the effects of honey bee stock and comb treatment on pathogen levels in mites and their pupal hosts. Tukey’s HSD post-hoc analyses were conducted to determine specific significant differences when appropriate. Further, Pearson correlation analyses were performed to examine the relationships between pathogen loads in wax and newly emerged bees collected from the same comb, and between pathogen loads in pupae and their infesting mites. Bonferroni correction was performed to adjust *p*-values for the multiple comparisons made. Pathogen-related analyses were conducted in JMP v12. 

## 3. Results

### 3.1. Effects of Honey Bee Stock and Comb Irradiation on Colony Performance

#### 3.1.1. Bee Population

**Figure 1:**
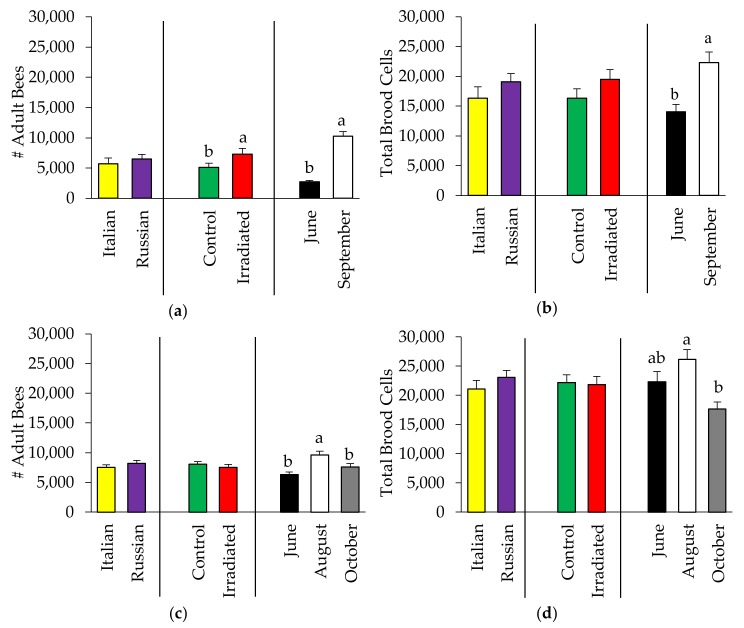
Bee and brood production (mean ± SE) in Italian and Russian honey bee colonies having irradiated or non-irradiated (control) combs: (**a**) 2015 adult bees; (**b**) 2015 brood cells; (**c**) 2016 adult bees; and (**d**) 2016 brood cells. For each group, bars with the same letters are not significantly different (*p* > 0.05); without letters indicates no differences.

Colony performance was measured by the strength of colonies (adult bee population and brood size), and the amount of stored pollen. For the bee population of the colonies, ANOVA showed no two-way interactions. Regardless of comb treatments, the strength of all colonies was consistently similar in both the Italian and Russian honey bee colonies for 2015 (Adult population: F = 0.84, *p* = 0.363, Figure 1a; Brood size: F = 1.70, *p* = 0.198, Figure 1b) and 2016 (Adult population: F = 0.56, *p* = 0.456, Figure 1c); Brood size: F = 0.70, *p* = 0.406, Figure 1d). While comb treatment did not affect the amount of brood (2015: F = 1.34, *p* = 0.252; 2016: F = 0.05, *p* = 0.818), its influence on adult bee population was inconsistent with colonies with irradiated combs being more populous in 2015 (F = 6.42, *p* = 0.014), but not in 2016 (F = 0.63, *p* = 0.430). As expected, all colonies continued to grow until the end of summer and beginning of fall (2015: F = 99.32, *p* < 0.0001 (Adult population); F = 9.26, *p* = 0.004 (Brood size)), and then declined thereafter as observed in 2016 (F = 9.76, *p* = 0.0002 (Adult population); F = 4.81, *p* = 0.011 (Brood size)). 

#### 3.1.2. Pollen Availability

**Figure 2:**
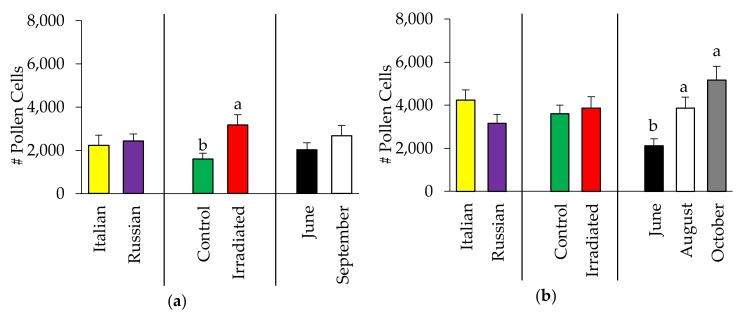
Amount (mean ± SE) of stored pollen in Italian and Russian honey bee colonies having irradiated or non-irradiated (control) combs in (**a**) 2015 and (**b**) 2016. For each group, bars with the same letters are not significantly different (*p* > 0.05); without letters indicates no differences.

Analysis of the amount of stored pollen showed no significant two-way interactions. Likewise, the amount of stored pollen did not vary between the Italian and Russian honey bee colonies both in 2015 (F = 2.30, *p* = 0.135; Figure 2a) and 2016 (F = 2.65, *p* = 0.108; Figure 2b). However, irradiation of combs affected the amount of stored pollen in 2015 (F = 7.39, *p* = 0.009), but not in 2016 (F = 0.31, *p* = 0.581). Further, the amount of pollen stored in 2015 colonies was the same for both months of observations (F = 0.01, *p* = 0.905), while there was an increasing amount of stored pollen through time in 2016 (F = 8.83, *p* = 0.0004). 

### 3.2. Effects of Honey Bee Stock and Comb Irradiation on Varroa Population

#### Colony Mites

**Figure 3:**
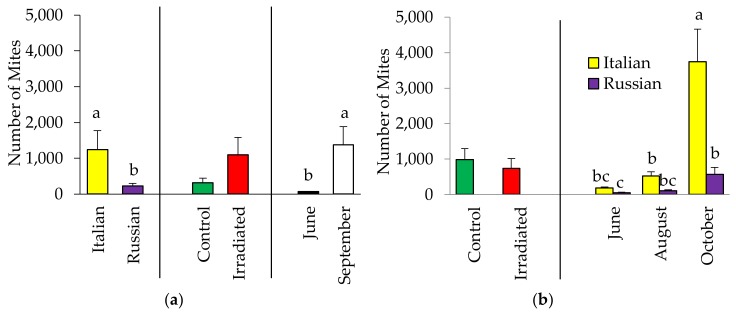
Total (mean ± SE) number of *Varroa* in Italian and Russian honey bee colonies having irradiated or non-irradiated (control) combs in (**a**) 2015 and (**b**) 2016. For each group, bars with the same letters are not significantly different (*p* > 0.05); without letters indicates no differences.

For the population of mites in the colonies in 2015, no significant two-way interactions or comb irradiation effect (F = 3.59, *p* = 0.064) were observed. However, a significant effect of honey bee stock was detected with the Italian colonies supporting more mites than the Russian colonies (F = 7.87, *p* = 0.007, Figure 3a). Additionally, mite population significantly increased in September (F = 24.35, *p* < 0.0001). Similarly, in 2016, no effect of comb-irradiation was detected (F = 0.16 *p* = 0.694). However, a significant interaction between stock and month of observation was recorded (F = 22.38, *p* < 0.0001, Figure 3b). The highest mite population (3,738 mites) was recorded in the Italian colonies in October. Mite populations in the Russian colonies were generally low having less than 600 mites in October, but was similar to the mite populations observed in the Italian colonies in June and August, and in Russian colonies in August. The lowest mite population was observed in June in the Russian colonies, but was comparable to that observed in Russian colonies in August. For specific information regarding brood and adult infestation levels separately, see Figures S1 and S2.

### 3.3. Effects of Honey Bee Stock and Comb Irradiation on Pathogen Load

#### 3.3.1. Pathogens Detected in Wax

A total of 81 composite wax samples from Italian (control = 9; irradiated = 5) and Russian (control = 7; irradiated = 6) colonies were collected in June, August and October 2016. Only wax samples derived from comb that contained brood during the experiment were used for this analysis. Of the 81 wax samples, only three were positive for *Nosema*. All samples were negative for *A. apis* or *P. larvae*. All but one sample was positive for at least one of the 10 pathogens investigated in this study (see Table S3 for average prevalence of each pathogen by sample type).

**Figure 4:**
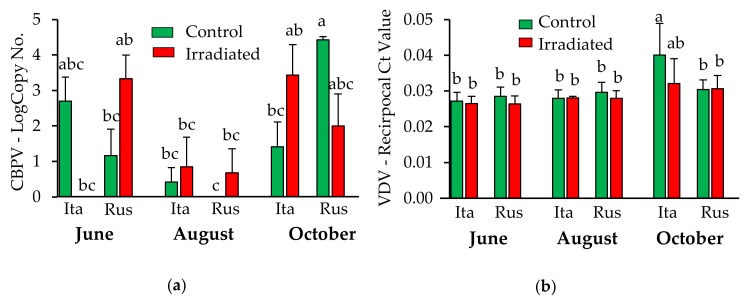
Wax-borne levels (mean ± SE) of (**a**) Chronic bee paralysis virus (CBPV) and (**b**) Varroa destructor virus-1 (VDV-1) in Italian and Russian honey bee colonies having irradiated or non-irradiated (control) combs across the season. Bars with the same letters are not significantly different (*p* > 0.05). Ita = Italian; Rus = Russian

Analysis of variance indicated that sampling time significantly influenced levels of EFB, AKI, BQCV, DWV, LSV-U, and the total number of pathogens detected (*p* < 0.002 for each pathogen; see Figure S3). For CBPV and VDV-1 levels, there was a three-way interaction among sampling time, comb treatment and honey bee stock. For CBPV, overall, August levels were lower than June and October. The increase in October appears to be driven by the increase of CBPV levels in the Russian control colonies only (F = 7.84, *p* = 0.003; Figure 4a). In October, VDV-1 levels were significantly higher in Italian control comb as compared to all treatment groups, except for Italian irradiated comb which was intermediate but not significantly different from any of the other treatments (F = 4.66, *p* = 0.02; Figure 4b). Otherwise, no comb irradiation or honey bee stock effects on the pathogen levels in wax were detected.

#### 3.3.2. Pathogens Detected in Newly Emerged Bees

**Figure 5:**
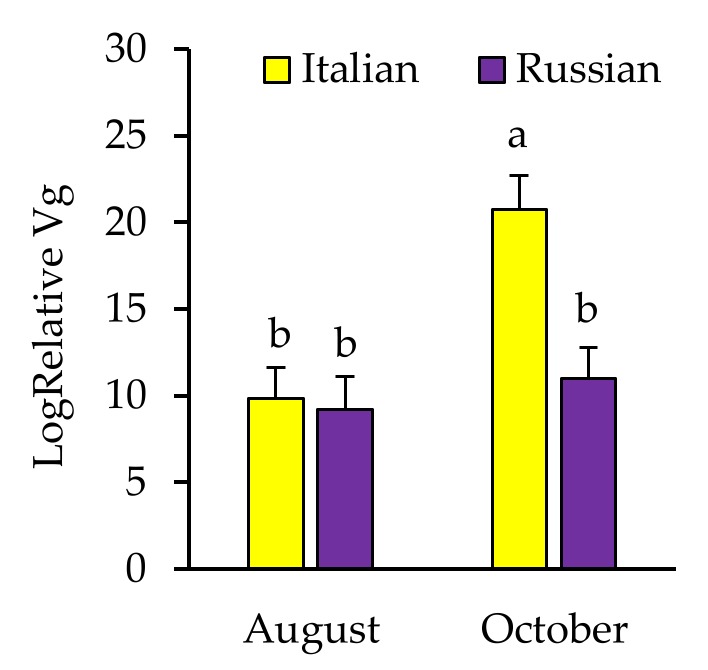
Relative amount (mean ± SE) of *Vg* (the gene encoding for the yolk-precursor protein, vitellogenin) in newly emerged bees from Italian and Russian colonies in August and October 2016. Bars with the same letters are not significantly different (*p* > 0.05).

Pools of 12 newly emerged bees sampled in August and October 2016 were analyzed for pathogen loads and levels of the gene encoding for the yolk-precursor protein, *Vg*, with samples examined from 10 Italian control, 9 Italian irradiated, 10 Russian control, and 9 Russian irradiated colonies. Few bee samples were positive for AKI, LSV, or EFB, while none were positive for *A. apis*, *P. larvae*, or *Nosema* spp. Thus, these limited data were not statistically analyzed for effects of irradiation or stock. In terms of how all of these factors may have influenced general robustness of the newly emerged bees, *Vg* results indicated a significant interaction between honey bee stock and sampling time. Overall, *Vg* increased from August to October in Italian colonies, but levels remained the same over time for Russian bees (F = 5.956, *p* = 0.02; Figure 5). 

**Figure 6:**
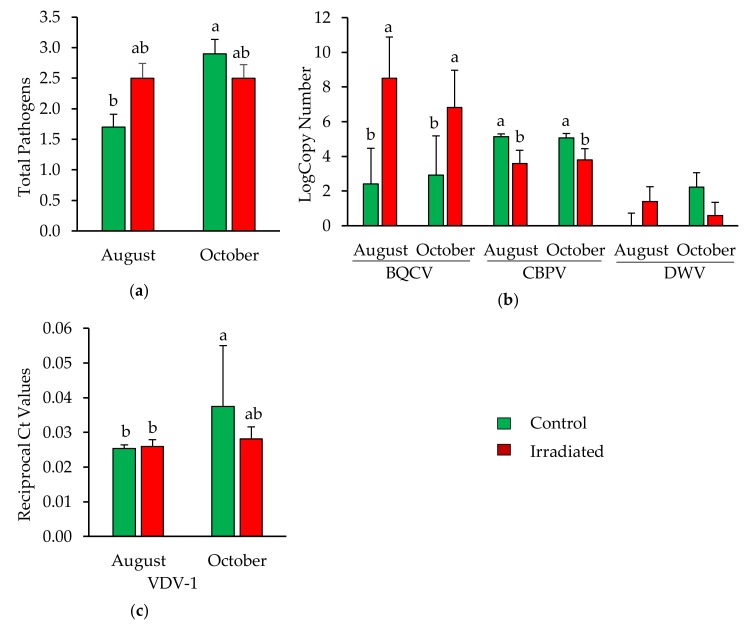
Pathogen loads for newly emerged bees from colonies having irradiated or non-irradiated (control) combs in August and October 2016: (**a**) Total number (mean ± SE) of pathogens detected; (**b**) levels (mean ± SE) of Black queen cell virus (BQCV), CBPV and Deformed wing virus (DWV); and (**c**) VDV-1. Bars without letters are not significantly different (*p* > 0.05).

ANOVA revealed a significant two-way interaction between sampling time and comb treatment on the number of pathogens detected in newly emerged bees (F = 6.54, *p* = 0.016; Figure 6a). For the levels of DWV, no influence of sampling time (*p* = 0.80), honey bee stock (*p* = 0.17) or comb irradiation (*p* = 0.17) was detected (Figure 6b). While the levels of BQCV increased (F = 5.11, *p* = 0.03), a decrease of CBPV levels (F = 7.42, *p* = 0.01) was observed in bees reared on irradiated combs (Figure 6b). For VDV-1, an interaction between month and comb treatment was observed (*p* = 0.049; Figure 6c). The analyses indicated that VDV-1 levels and the total number of pathogens detected significantly increased in bees reared in control comb, while there was no significant change in bees reared in irradiated comb.

**Figure 7:**
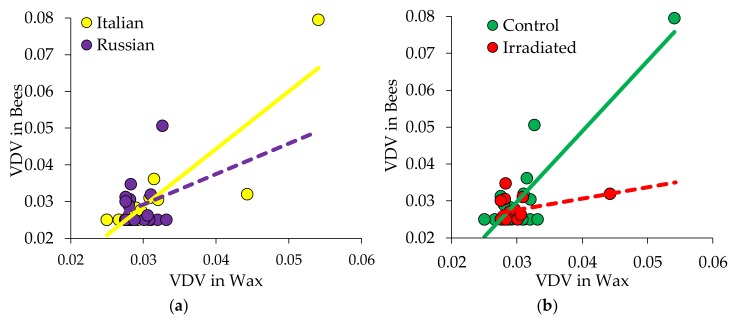
VDV-1 levels were positively correlated for newly emerged bees and wax collected from the same comb for samples from: (**a**) Italian colonies (yellow, solid line), but not for samples from Russian colonies (purple, dashed line), and (**b**) colonies with control (green, solid line) combs, but not from colonies with irradiated (red, dashed line) combs.

Subsequent analysis of the possible relationship between the wax in which the bees were reared and the bees’ ultimate pathogen load showed a high correlation between the levels of VDV-1 in wax and the bees that emerged from the comb for Italian colonies, but not for Russian colonies, regardless of comb treatment (r = 0.858, *p* < 0.0001 for Italian; *p* = 0.67 for Russian; Figure 7a). Overall, there was a high correlation between wax and newly emerged bees for the control combs (r = 0.73, *p* = 0.003; Figure 7b), but not for irradiated combs (*p* = 0.118) for the levels of VDV-1. Refer to Table S4 for full correlation analyses not discussed here as there were no other significant relationships observed.

#### 3.3.3. Pathogens Detected in Pupae and Their Infesting *Varroa*

**Figure 8:**
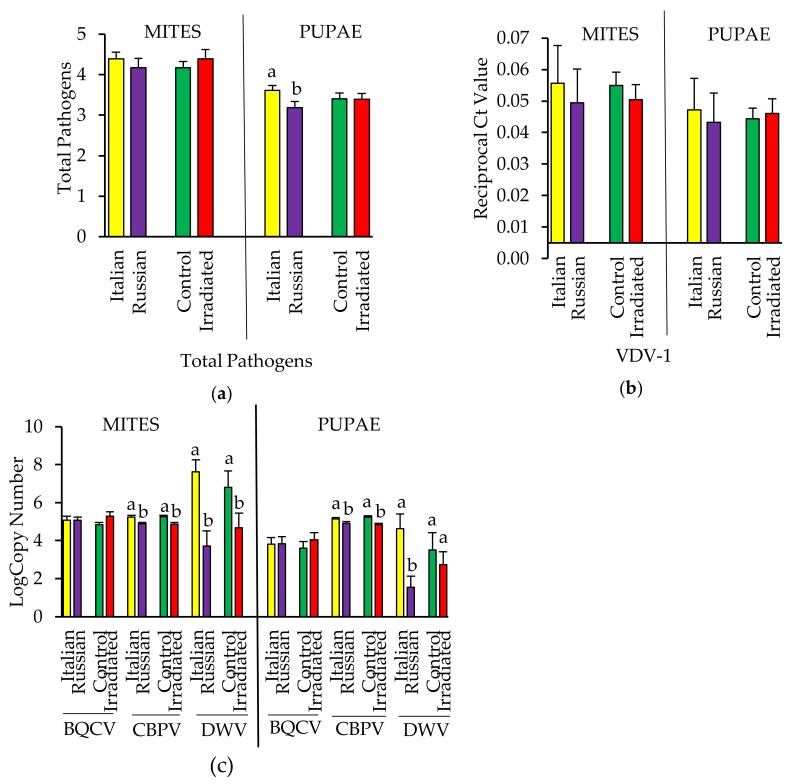
(**a**) Total number (mean ± SE) of pathogens detected; (**b**) levels (mean ± SE) of VDV-1 and (**c**) other viruses in mites and their pupal hosts collected from Italian and Russian colonies having either irradiated or non-irradiated (control) combs. For each group, bars with the same letters are not significantly different (*p* > 0.05); without letters indicates no differences.

In October 2016, two to three mite-infested pupae along with the infesting foundress *Varroa* were collected from three colonies from each treatment group, resulting in eight to nine individual *Varroa* and pupal samples per treatment. All viruses tested were detected in at least some mites and pupae (see Table S1), and pupae from Italian colonies had a higher number of detections overall (F = 4.73, *p* = 0.037; Figure 8a). AKI was only detected in two pupae and 16 out of 44 mites, and so was not considered in analyses other than that of total pathogens detected. There were no effects of comb irradiation or honey bee stock on levels of VDV-1 (Figure 8b) or BQCV (Figure 8c). However, viral analysis of the infesting *Varroa* indicated a significant effect of honey bee stock for DWV with mites collected from Italian colonies having higher viral loads as compared to those mites collected from Russian colonies (F = 16.19, *p* = 0.003, Figure 8c). Analysis of the infested pupal hosts showed the same effect (F = 9.36, *p* = 0.005, Figure 8c). For CBPV, there was a significant effect of comb irradiation detected for both pupae and their infesting mites with individuals from control colonies having higher levels than those from irradiated colonies (mites: F = 13.44, *p* = 0.0009; pupae: F = 15.25, *p* = 0.0005; Figure 8c). Further, mites and pupae collected from the Italian colonies also had increased CBPV titers as compared to those from Russian colonies (mites: F = 11.02, *p* = 0.002; pupae: F = 4.67, *p* = 0.039; Figure 8c) with no interaction between stock and comb treatment for either. 

An examination of the relationship between viral levels in the *Varroa* and their pupal hosts showed that mite and pupal VDV-1 and CBPV levels were highly correlated, regardless of honey bee stock or comb treatment (see Table 1). For DWV, mite and pupal host levels were also correlated overall, but this was driven largely by samples from Italian colonies and those with non-irradiated combs (Table 1). After Bonferroni correction, no significant correlations for BQCV or total pathogens in mites and pupae were detected. 

#### 3.3.4. *Nosema* Spore Count

**Figure 9:**
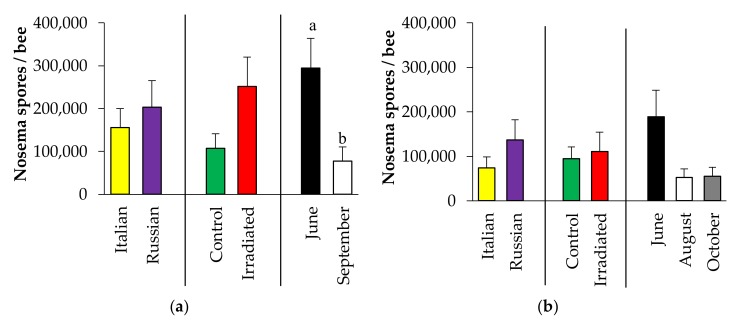
Number of *Nosema* spores (mean ± SE) in Italian and Russian honey bee colonies having irradiated or non-irradiated (control) combs: (**a**) 2015 and 2016 (**b**). For each group, bars with different letters are significantly different (*p* < 0.05); without letters indicates no differences.

For both years of observation, ANOVA revealed no significant two-way interactions, no significant influences of honey bee stocks (2015: F = 0.60, *p* = 0.443; 2016: F = 2.07, *p* = 0.153), and comb-irradiation (2015: F = 2.33, *p* = 0.134; 2016: F = 0.00, *p* = 0.947). On average, *Nosema* spore counts were generally low (2015: Italian = 155, 903 ± 44,633 spores, Russian = 203,333 ± 62,438 spores (Figure 9a); 2016: Italian = 74,107 ± 25,056 spores, Russian = 136,458 ± 45,704 spores (Figure 9b)). In 2015, sampling time affected *Nosema* spore counts with higher counts recorded in June than in September (F = 7.01, *p* = 0.011), but did not vary among months of observation in 2016 (F = 2.88, *p* = 0.061) (Figure 9a,b). 

### 3.4. Effects of Honey Bee Stock and Comb Irradiation on Colony Survival 

**Figure 10:**
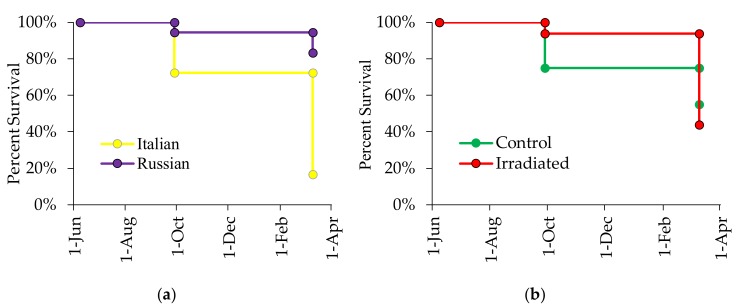
Effects of honey bee stock (**a**) and comb irradiation (**b**) on colony survival in 2015.

Survival analysis showed that Russian honey bee colonies lived longer than Italian honey bee colonies. In the 2015 experiment, 83% of the Russian honey bee colonies were still alive in March 2016 while only 17% of the Italian colonies survived (χ^2^ = 14.85, *p* = 0.0001; Figure 10a). Comb-irradiation did not improve survival of these colonies (χ^2^ = 0.04, *p* = 0.851; Figure 10b). There were 30 colony survivors by the end of observation in September 2015 (Russian Control: 10, Russian Irradiated: 7; Italian Control: 5, Italian Irradiated: 8). Of these, 18 colonies (Russian Control: 9, Russian Irradiated: 6; Italian Control: 2, Italian Irradiated: 1) survived the winter. 

**Figure 11:**
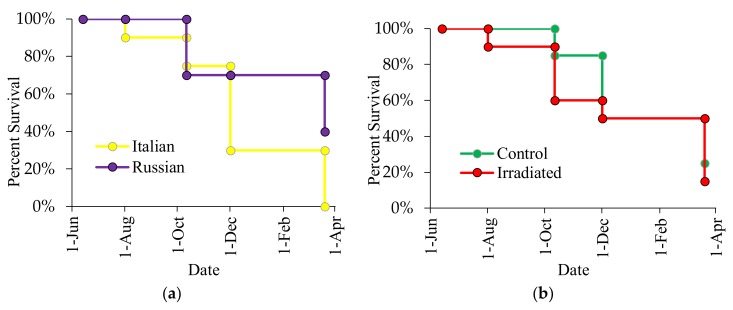
Effects of honey bee stock (**a**) and comb irradiation (**b**) on colony survival in 2016.

The same trend was observed during the 2016 experiment. By March 2017, all Italian colonies had died whereas 40% of the Russian colonies were still alive (χ^2^ = 9.79, *p* = 0.002; Figure 11a), seven of which were still alive in June 2017 (four control, three treated). No influence of comb irradiation on colony survival was also detected (χ^2^ = 0.54, *p* = 0.463; Figure 11b). The abrupt decline in colony survival was observed in October 2016 due to a historic flooding in the area about 2 weeks after the colony evaluation in August 2016. Although adult bees of these flooded colonies (one Italian and six Russians) moved up the top boxes, they eventually succumbed to small hive beetle infestations that attacked the decomposing brood at the bottom brood chamber as flood water slowly receded. There were 29 colony survivors in October 2016 (Russian Control: 8, Russian Irradiated: 6; Italian Control: 9, Italian Irradiated: 6). Although there were 14 Russian colonies (range adult infestation = 0.4% to 5.9%) and 15 Italian colonies (range adult infestation = 0.3% to 55%) alive during the observations in October 2016, no Italian colony survived in March 2017, likely due to high *Varroa* parasitism with the exception of two colonies that died even though they had levels below the economic treatment threshold. In contrast, eight Russians (five control, three irradiated) colonies overwintered successfully (March 2017), seven of which were still alive in June 2017 despite having a range of 0.6% to 3.6% adult infestation in October 2016. 

## 4. Discussion

This study demonstrates that honey bee genotype largely influences *Varroa* population in *A. mellifera* colonies. For both 2015 and 2016, the Russian honey bee colonies supported lower number of *Varroa* and thus, overwintered more successfully confirming earlier studies [7,15,50,51,52]. 

Comb irradiation consistently showed no influence in three of the five colony performance and mite infestation parameters measured during the two years of observation. The only significant influence of comb irradiation was observed in 2015 that resulted in increased adult bee population, and pollen stores in colonies with irradiated combs. However, this difference did not occur in 2016. Thus, the increase in adult bee population in colonies with irradiated combs in 2015 was probably associated with increased pollen availability since pollen is required for reproduction and development [53]. Nevertheless, no correlation between adult bees and stored pollen, neither brood nor stored pollen has been reported [54]. Perhaps, bees consumed more of freshly collected pollen than stored pollen. Bees are known to prefer freshly collected pollen over stored pollen even if they have the same nutritional values [55]. Despite the increase in pollen stores in colonies with irradiated combs, much of the Italian honey bee colonies succumbed to high *Varroa* infestations. Dilution effect brought by the larger number of adult bees in colonies with irradiated combs would have reduced the number of phoretic mites in 2015. Yet, the overall adult mite infestation in colonies with irradiated combs was remarkably high, which was likely influenced by the high adult mite infestations in the Italian colonies with irradiated combs (up to 45% on adult bees in September 2015). The contrasting results on adult bee population, and amount of stored pollen between 2015 and 2016 may be due to several factors including colony strength and environmental factors, which can affect foraging activities of bees. 

The presence of *Varroa* can influence virus levels in the colony. However, mite-borne viruses occur in bee colonies even in seemingly healthy bees [56]. DWV has been detected in *Varroa*-free areas in Hawaii [57]. Indeed, our earlier study showed that mite-free bees have DWV, but those reared in irradiated combs had lower levels than those reared in non-irradiated combs [37]. In this study, we found a more pronounced influence of honey bee genotype on virus levels as compared to comb irradiation. Both *Varroa* and their pupal hosts from the Russian colonies had reduced DWV levels compared to those from Italian colonies. This is particularly interesting because all colonies were established from the same large package of bees and mites. Whether or not there are some physiological or biochemical process that limit DWV replication in Russian honey bees, as has been previously suggested [58], warrants continued study. An alternative explanation for the reduction in viruses in mites and pupae could potentially involve increased removal of virus-infected brood [59,60]. Russian bees are known for exhibiting both a high level of expression of general hygienic behavior, the removal of diseased brood [61], in addition to their increased VSH (*Varroa* sensitive hygiene) activity [62,63]. Highly hygienic Russian bees are also high groomers [63,64]. Hence, it is possible that mite and brood removal activities significantly lower both the number of mites and the highly virus-infected mites available for brood infestation thus, limiting viral replication in the bees and mites in Russian bee colonies. Further, levels of DWV, BQCV and the total number of pathogens detected were highly correlated between the *Varroa* and their host pupae collected from Italian colonies, but not from the Russian colonies. These findings highlight the possible influence of mite-resistant stock on viral loads in mites and their pupal hosts and further study on the mechanisms are necessary. Another potentially interesting aspect of differences in physiology between Russian and Italian bees was indicated by the vitellogenin findings. While *Vg* increased in bees collected from Italian colonies from August to October, as expected as the overwintering bee population is produced [65], this increase was absent in Russian bees. This could indicate that *Vg* may not be regulated in the same way in this stock and potentially other races that are more evolutionarily derived from colder climates. Impacts and repeatability of this result will be explored.

Another area of research that needs to be explored more fully is the transmission of wax-borne pathogens, particularly viruses. Honey bee combs are known to harbor pathogens harmful to honey bees such as AFB and chalkbrood [20]. Viruses are also claimed to permeate wax held in an incubator from either contact with virus-infected bees or through aerosolization [22]. Overall, about four viruses or pathogens were detected in wax samples with much of the samples being positive for AKI (73%) and VDV-1 (79%). For AKI, this did not translate into a high level of detections in newly emerged bees as only two pools from the Italian control colonies were positive for AKI. However, ~50% of the newly emerged bee samples were positive for VDV-1. The finding that VDV-1 only significantly increased both in wax and newly emerged bees from control combs provides some suggestive evidence that wax could serve as a viral reservoir and be a source for future infection. In addition, ~60% of wax samples had CBPV, and CBPV levels were lower in newly emerged bees, mites and their host pupae collected from irradiated combs than those from the control group. This indicates that the very minimal, but significant effect of reducing the replication rate of CBPV in pupae injected with an irradiated solution of the virus might be biologically relevant, despite it not having an effect on pupal mortality in our previous study [34]. Results here do, however, confirm that comb irradiation is not likely a useful treatment to reduce BQCV infection. Further, the presence of EFB (46%) in wax may also affect future bacterial infection. Being that this is the first study to document viral levels found in used honey bee comb, the impacts of wax-borne viruses on honey bee health are, as yet, unclear and require further investigation.

In terms of other pathogens, gamma irradiation is also known to inactivate *Nosema* spores, resulting in almost completely reduced germination and infectivity [34]. However, this does not appear to translate to reduced *Nosema* in field colonies, possibly due to the fact that in this case the comb may not have been the source of exposure. While no significant influence of honey bee stock was detected, a wide variation among colonies was recorded. The highest *Nosema* counts were observed in June (when the experiment commenced in 2015), which corroborates earlier findings [66]. This increase in *Nosema* spores may be associated with bees consuming more food as the colonies grew in spring. It has been reported that *Nosema* spores can be transmitted via food exchange [67], and that pollen feeding causes rapid establishment or high intensities of *Nosema* spores [68]. In 2015, 58% of the Italian colonies were infected with *Nosema* as compared to 71% in the Russian colonies. Of the infected Russian honey bee colonies, ~13% had more than 1 million spores all of which had irradiated combs. These colonies raised the estimates of average *Nosema* spores per bee in the Russian honey bees. None of the Italian colonies had 1 million *Nosema* spores during this time. Nevertheless, these highly infected Russian colonies were able to grow and survive, which was also observed in our earlier study [69]. This observation supports the claim that some Russian bees may display tolerance or resistance to *N. ceranae* [70]. Similarly, the majority of colonies had below the commonly accepted *Nosema* economic threshold level of 1 × 10^6^ in September 2015 and October 2016 (or at the end of observations), which may be too low for negative effects to be apparent [69]. Although *Nosema* counts are higher in June, they were lower from August to September, which may be associated with decreasing temperature as Fall approaches [71]. Nevertheless, analysis of *Nosema* via real-time PCR had zero detections in newly emerged bees, and only three of 81 wax samples were positive for *Nosema* spp., indicating that at least at the time of sampling *Nosema* has not spread via fecal deposits on the comb. 

While *N. ceranae* infection is presumed harmful in mite-resistant Russian bees [69], their survival was significantly higher than the Italian colonies with the absence of *Nosema* treatment. For 2015, 75% of the Russian honey bee colonies survived the following spring (March 2016) as compared to only 15% in the Italian colonies. For 2016, all of the Italian colonies had died by March 2017 while eight Russian honey bee colonies overwintered through March 2017, seven of which were still alive in June 2017. Although pollen nutrition increases the survival of both healthy and *Nosema*-infected bees [72], pollen cannot replace the loss or alteration of haemolymph protein as a consequence of mite feeding [73]. Pollen is also essential in brood production. Nevertheless, the increased pollen stores recorded in colonies with irradiated combs did not help Italian colonies with irradiated combs overcome the effects of injuries inflicted by *Varroa* and *Varroa*-transmitted viruses. This study confirms existing assertion that *Varroa* is still the number one problem of honey bees [74]. Although comb irradiation can reduce levels of some viruses, comb irradiation does not regulate populations of *Varroa*, which is a major vector of honey bee viruses. Thus, the use of mite-resistant stock is more helpful in regulating mite numbers in *A. mellifera* colonies. The interactive effects of comb irradiation and stock, namely that virus levels in newly emerged bees and mite-infested pupae can be decoupled from levels in wax or *Varroa*, provide valuable insights into how different management strategies can work in concert. 

## 5. Conclusions

There are several factors that determine productivity and survival of colonies including bee genotypes, and pressure from pathogen and parasite exposures. While both Italian and Russian bees were very similar in colony performance in this study, their responses to both mites and some viruses vary. The mite-resistant Russian honey bees showed some degree of resistance to DWV and CBPV, which may add to the value of this stock. The contribution of irradiating comb in improving honey bee health and survival appears to be subtle. However, comb irradiation may be useful as part of an integrated pest management strategy with the addition of using mite-resistant stocks.

## Figures and Tables

**Table 1:** Summary of correlation analyses for viral and total pathogen levels in *Varroa* and their respective infested pupae. With Bonferroni correction, a *p*-value < 0.006 indicates significance as indicated in bold type.

Treatment Type	VDV1	DWV	BQVC	CBPV	Total Pathogens Detected
Control	**r = 0.897,** ***p* < 0.0001**	**r = 0.674,** ***p =* 0.003**	r = 0.173, *p =* 0.508	r = 0.312, *p =* 0.222	r = 0.122, *p =* 0.642
Irradiated	**r = 0.833,** ***p* < 0.0001**	r = 0.585, *p =* 0.011	r = 0.404, *p =* 0.096	r = 0.285, *p =* 0.251	r = 0.324, *p =* 0.189
Italian	**r = 0.922,** ***p* < 0.0001**	**r = 0.687,** ***p =* 0.002**	r = 0.475, *p =* 0.046	r = 0.352, *p =* 0.152	r = 0.121, *p =* 0.631
Russian	**r = 0.737,** ***p* < 0.0001**	r = 0.324, *p =* 0.204	r = 0.180, *p =* 0.489	r = 0.534, *p =* 0.024	r = 0.255, *p =* 0.323
Italian, Control (*n* = 9)	**r = 0.968,** ***p* < 0.0001**	r = 0.802, *p =* 0.009	r = 0.413, *p =* 0.269	r = −0.456, *p =* 0.218	r = −0.791, *p =* 0.011
Italian, Irradiated (*n* = 9)	**r = 0.865,** ***p =* 0.003**	r = 0.829, *p =* 0.006	r = 0.527, *p =* 0.145	r = −0.034, *p =* 0.932	r = 0.661, *p =* 0.052
Russian, Control (*n* = 8)	r = 0.781, *p =* 0.022	r = 0.574, *p =* 0.137	r = −0.084, *p =* 0.843	r = 0.236, *p =* 0.574	r = 0.535, *p =* 0.172
Russian, Irradiated (*n* = 9)	**r = 0.835,** ***p =* 0.005**	r = −0.083, *p =* 0.831	r = 0.245, *p =* 0.525	r = 0.816, *p =* 0.007	r = 0.124, *p =* 0.751
Overall (*n* = 35)	**r = 0.846,** ***p* < 0.0001**	**r = 0.636,** ***p* < 0.0001**	r = 0.350, *p =* 0.039	**r = 0.492,** ***p =* 0.003**	r = 0.236, *p =* 0.172

## Supplementary Materials

The following are available online at http://www.mdpi.com/2075-4450/10/1/15/s1, Table S1: Primer information, Table S2: Pathogen prevalence, Table S3: Summary of the correlation analyses for newly emerged bees and wax, Figure S1: Adult bee *Varroa* infestation, Figure S2: Brood *Varroa* infestation, and Figure S3: Levels of all wax-borne pathogens based on season, stock and comb treatment.
