# Supplementary material for: Comb Irradiation Has Limited, Interactive Effects on Colony Performance or Pathogens in Bees, Varroa destructor and Wax Based on Two Honey Bee Stocks
Source: Insects. 2019 Jan 8;10(1):15. doi: 10.3390/insects10010015 (PMC6359134; doi:10.3390/insects10010015)
Supplement: Supplementary file 1 [file insects-10-00015-s001.pdf]

Article

Comb Irradiation Has Limited, Interactive Effects on Colony Performance or Pathogens in Bees, *Varroa destructor* and Wax Based on Two Honey Bee Stocks

Lilia I. de Guzman *, Michael Simone-Finstrom *, Amanda M. Frake and Philip Tokarz

Honey Bee Breeding, Genetics and Physiology Laboratory, USDA-ARS, Baton Rouge, LA 70820, USA; mandy.frake@ars.usda.gov (A.M.F.); philip.tokarz@ars.usda.gov (P.T.)

* Correspondence: lilia.deguzman@ars.usda.gov (L.I.d.G.); michael.simonefinstrom@ars.usda.gov (M.S.-F.); Tel.: +1-225-767-9282 (L.I.d.G.)

Received: 28 September 2018; Accepted: 8 December 2018; Published: date

Table S1. Primer information.

	fwd seq	rev seq	Reference
AKI	CTT TCA TGA TGT GGA AAC TCC	AAA CTG AAT AAT ACT GTG CGT A	Francis & Kryger 2012
A. apis	TCT GGC GGC CGG TTA AAG GCT TC	GTT TCA AGA CGG GCC ACA AAC	Evans *et al.* 2006
EFB	TGT TGT TAG AGA AGA ATA GGG GAA	CGT GGC TTT CTG GTT AGA	
LSV-Univ	CGT GCG GAC CTC ATT TCT TCA TGT	CTG CGA AGC ACT AAA GCG TT	Daughenbaugh *et al.* 2015
Nosema-Both	AGC AGC CGC GGT AAT ACT TGT TC	GTT CGT CCA GTC AGG GTC GT	Alburaki *et al.* 2018
P. larvae RPS18	TTC ACG GCT AAC AAA ATT AAA CA	TTC GCA GAA GTT CCG GTT AC	Boncristiani *et al.* 2012
VDV-1 CP	CTG TAG TTA AGC GGT TAT TAG AA	GGT GCT TCT GGA ACA GCG GAA	Ryabov *et al.* 2017
BQCV	TCG CAG AGT TCC AAA TAC CG	TAT CAT CTC CCG CAC CTA CC	Yoo *et al.* 2008
CBPV	CGC AAG TAC GCC TTG ATA AAG AAC	ACT ACT AGA AAC TCG TCG CTT CG	Blanchard *et al.* 2007
DWV-1	GAG ATT GAA GCG CAT GAA CA	TGA ATT CAG TGT CGC CCA TA	Boncristiani *et al.* 2012
Pros54	TCG AAC CAA GAT GGT ACT GGA A	TTG TTG TGC TTG CAG TCG TG	Cameron *et al.* 2013
VgMC	AGT TCC GAC CGA CGA CGA	TTC CCT CCC ACG GAG TCC	Evans *et al.* 2006

Table S2. ANOVA parameters for main effects and associated interactions for adult population, total brood cells and mite population in 2015 and 2016.

Year	Effect	Adult Population		Total Brood Cells		Mite Population	
		F	*P*	F	*P*	F	*P*
2015	Date	99.32	< 0.0001	9.26	0.003	24.35	< 0.0001
	Stock	0.84	0.362	1.70	0.198	7.87	0.007
	Date*Stock	0.04	0.836	0.24	0.625	3.47	0.068
	Treatment	6.42	0.014	1.34	0.252	3.59	0.063
	Date*Treatment	0.05	0.822	3.86	0.054	2.26	0.138
	Stock*Treatment	0.02	0.876	0.01	0.921	1.63	0.207
	Date*Stock*Treatment	0.03	0.857	1.07	0.305	1.65	0.205
2016	Date	9.76	0.0002	4.81	0.011	21.05	< 0.0001
	Stock	0.56	0.456	0.70	0.406	22.38	< 0.0001
	Date*Stock	1.55	0.22	0.22	0.806	4.21	0.018
	Treatment	0.63	0.43	0.05	0.818	0.16	0.694
	Date*Treatment	0.36	0.698	0.28	0.76	0.09	0.910
	Stock*Treatment	1.74	0.191	3.36	0.071	0.00	0.998
	Date*Stock*Treatment	2.53	0.086	0.52	0.597	0.02	0.982

Table S3. Pathogen prevalence based on sample type and treatment group.

% Infected per pathogen

	AKI				VDV-1 (DWV-B)				LSV-U			
	control		Irradiated		control		Irradiated		control		Irradiated	
**Wax** n=80	**72.9**		**71.9**		**81.3**		**78.1**		**25.0**		**18.8**	
	Ital	Rus	Ital	Rus	Ital	Rus	Ital	Rus	Ital	Rus	Ital	Rus
	81.5	61.9	64.3	77.8	77.8	85.7	85.7	72.2	22.2	28.6	14.3	22.2
**Newly Emerged** n=37	**10.0**		**0.0**		**40.0**		**52.9**		**0.0**		**5.6**	
	Ital	Rus	Ital	Rus	Ital	Rus	Ital	Rus	Ital	Rus	Ital	Rus
	20.0	0	0.0	0	40.0	40	55.6	50	0.0	0	11.1	0
**Mite** n=35	**23.5**		**55.6**		**100.0**		**100.0**		**11.8**		**11.1**	
	Ital	Rus	Ital	Rus	Ital	Rus	Ital	Rus	Ital	Rus	Ital	Rus
	11.1	37.5	44.4	66.7	100	100	100	100	22.2	0.0	11.1	11.1
**Pupae** n=35	**5.9**		**0.0**		**94.1**		**100.0**		**0.0**		**0.0**	
	Ital	Rus	Ital	Rus	Ital	Rus	Ital	Rus	Ital	Rus	Ital	Rus
	0.0	12.5	0.0	0.0	100	87.5	100	100	0.0	0.0	0.0	0.0

	BQCV				CBPV				DWV (DWV-A)			
	control		Irradiated		control		Irradiated		control		Irradiated	
**Wax** n=80	**37.5**		**34.4**		**60.4**		**56.3**		**60.4**		**65.6**	
	Ital	Rus	Ital	Rus	Ital	Rus	Ital	Rus	Ital	Rus	Ital	Rus
	40.7	33.3	21.4	44.4	63.0	57.1	64.3	50.0	51.9	71.4	64.3	66.7
**Newly Emerged** n=37	**60.0**		**100.0**		**100.0**		**94.1**		**15.0**		**17.6**	
	Ital	Rus	Ital	Rus	Ital	Rus	Ital	Rus	Ital	Rus	Ital	Rus
	50.0	70	####	100	####	100	####	87.5	10.0	20	22.2	12.5
**Mite** n=35	**100.0**		**100.0**		**100.0**		**100.0**		**82.4**		**72.2**	
	Ital	Rus	Ital	Rus	Ital	Rus	Ital	Rus	Ital	Rus	Ital	Rus
	100	100	100	100	100	100	100	100	100	62.5	88.9	55.6
**Pupae** n=35	**88.2**		**94.4**		**100.0**		**100.0**		**52.9**		**50.0**	
	Ital	Rus	Ital	Rus	Ital	Rus	Ital	Rus	Ital	Rus	Ital	Rus
	88.9	87.5	88.9	100	100	100	100	100	66.7	37.5	77.8	22.2

	EFB				Nosema				A. apis			
	control		Irradiated		control		Irradiated		control		Irradiated	
**Wax** n=80	**47.9**		**40.6**		**4.2**		**3.1**		**0.0**		**0.0**	
	Ital	Rus	Ital	Rus	Ital	Rus	Ital	Rus	Ital	Rus	Ital	Rus
	37.0	61.9	35.7	44.4	0.0	9.5	7.1	0.0	0.0	0.0	0.0	0.0
**Newly Emerged** n=37	**0.0**		**5.6**		**0.0**		**0.0**		**0.0**		**0.0**	
	Ital	Rus	Ital	Rus	Ital	Rus	Ital	Rus	Ital	Rus	Ital	Rus
	0.0	0	0.0	12.5	0.0	0	0.0	0	0.0	0	0.0	0
**Mite** n=35												
**Pupae** n=35												

	P. larvae				Average # of detections			
	control		Irradiated		control		Irradiated	
**Wax** n=80	**0.0**		**0.0**		**3.7 ± 1.7**		**3.6 ± 1.4**	
	Ital	Rus	Ital	Rus	Ital	Rus	Ital	Rus
	0.0	0.0	0.0	0.0	3.6 ± 1.7	3.9 ± 1.9	3.6 ± 1.2	3.6 ± 1.6
**Newly Emerged** n=37	**0.0**		**0.0**		**2.3 ± 0.8**		**2.5 ± 0.7**	
	Ital	Rus	Ital	Rus	Ital	Rus	Ital	Rus
	0.0	0	0.0	0	2.2 ± 0.9	2.3 ± 0.7	2.7 ± 0.7	2.3 ± 0.7
**Mite** n=35					**4 ± 0.6**		**4.0 ± 0.9**	
					Ital	Rus	Ital	Rus
					4.3 ± 0.5	4 ± 0.7	4.4 ± 0.8	4.3 ± 1.1
**Pupae** n=35					**3.0 ± 0.6**		**3.0 ± 0.6**	
					Ital	Rus	Ital	Rus
					3.5 ± 0.5	3.3 ± 0.7	3.7 ± 0.5	3.1 ± 0.6

Table S4. Summary of the correlation analyses for newly emerged bees and wax sampled from the same comb. With Bonferoni correction, a *p*-value < 0.006 indicates significance as indicated in bold type.

Treatment Type	VDV1	DWV	BQCV	CBPV	Total Pathogens Detected
Non-irradiated	**r = 0.730, p<.001**	r = -0.202, p=0.394	r = 0.201, p=0.395	r = 0.205, p=0.387	r = 0.246, p=0.295
Irradiated	r = 0.406, p=0.118	r = -0.230, p=0.391	r = 0.039, p=0.887	r = 0.301, p=0.258	r = 0.070, p=0.798
Italian	**r = 0.858, p<.0001**	r = -0.182, p=0.456	r = 0.054, p=0.825	r = -0.084, p=0.731	r = 0.306, p=0.202
Russian	r = 0.110, p=0.675	r = -0.256, p=0.321	r = 0.041, p=0.875	r = 0.322, p=0.207	r = 0.076, p=0.771
Italian, non-irradiated (n=10)	**r = 0.910, p<.001**	r = -0.167, p=0.646	r = 0.383, p=0.275	r = 0.388, p=0.268	r = 0.382, p=0.277
Italian, irradiated (n=9)	**r = 0.842, p=0.004**	r = -0.189, p=0.626	r = -0.208, p=0.592	r = 0.081, p=0.836	r = 0.046, p=0.906
Russian, non-irradiated (n=10)	r = 0.248, p=0.489	r = -0.249, p=0.487	r = -0.072, p=0.842	r = -0.121, p=0.740	r = 0.059, p=0.871
Russian, irradiated (n=7)	r = -0.479, p=0.277	r = -0.258, p=0.576	r = 0.505, p=0.247	r = 0.566, p=0.8185	r = 0.108, p=0.818

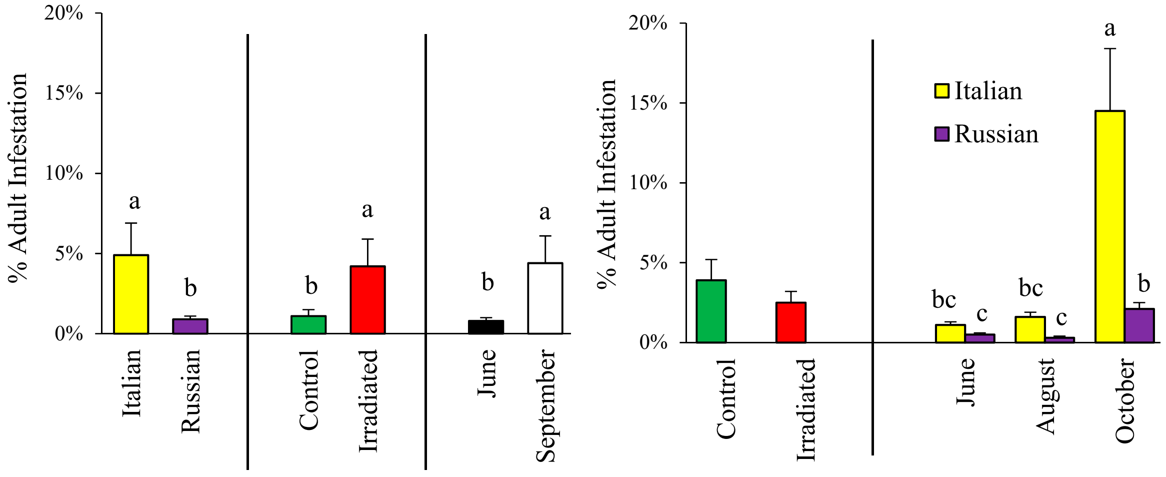

(**a**) (**b**)

Figure S1. Prevalence (mean ± SE) of *Varroa* on adult bees in Italian and Russian honey bee colonies having irradiated or non-irradiated (control) combs in (**a**) 2015 and (**b**) 2016. For each group, bars with the same letters are not significantly different ($p > 0.05$); without letters indicates no differences.

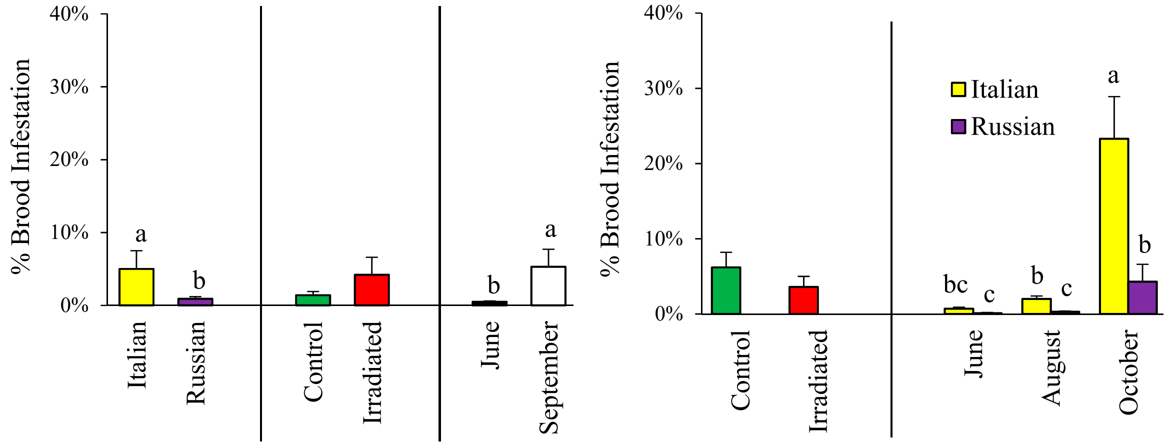

(**a**) (**b**)

Figure S2. Prevalence (mean ± SE) of *Varroa* in sealed brood of Italian and Russian honey bee colonies having irradiated or non-irradiated (control) combs in (**a**) 2015 and (**b**) 2016. For each group, bars with the same letters are not significantly different ($p > 0.05$); without letters indicates no differences.

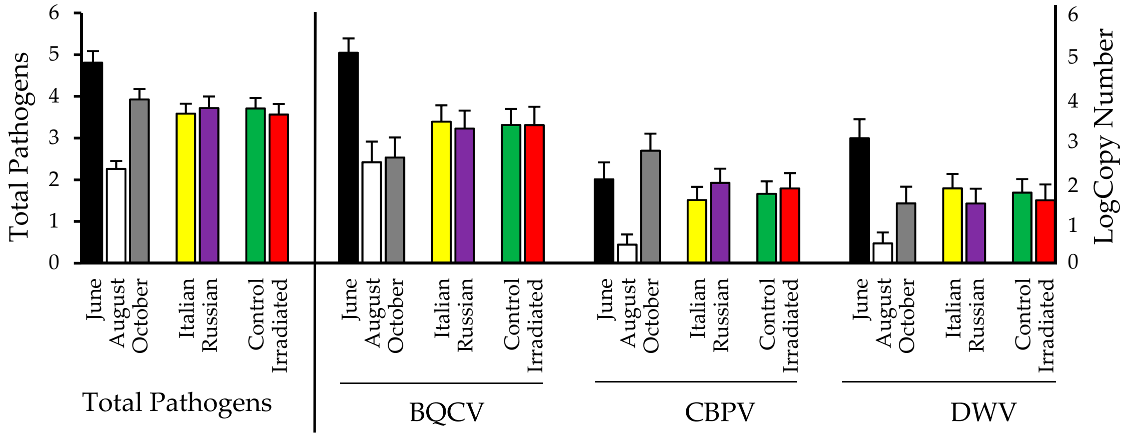

(**a**)

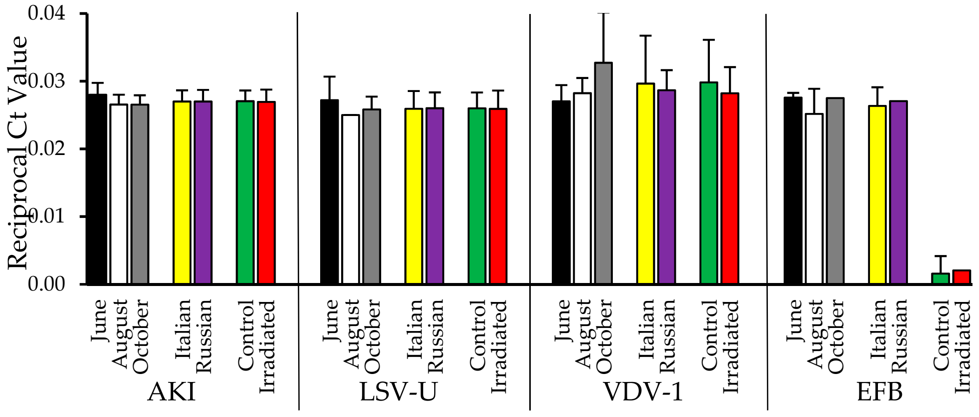

(b)

Figure S3. Levels (mean ± SE) of wax-borne pathogens by season, stock and comb treatment (control or irradiated)
